# Antimicrobial activity of supramolecular salts of gallium(III) and proflavine and the intriguing case of a trioxalate complex

**DOI:** 10.1038/s41598-022-07813-0

**Published:** 2022-03-07

**Authors:** Marzia Guerrini, Simone d’Agostino, Fabrizia Grepioni, Dario Braga, Andrii Lekhan, Raymond J. Turner

**Affiliations:** 1 Dipartimento di Chimica “Giacomo Ciamician”, Università Di Bologna, Via Selmi, 2, 40126 Bologna, Italy; 2 Department of Biological Sciences, University of Calgary, 2500 University Drive NW, Calgary, AB T2N 1N4 Canada

**Keywords:** Crystal engineering, Antimicrobials, X-ray diffraction

## Abstract

The use of the gallium oxalate complex [Ga(ox)_3_]^3−^ as a building block in the formation of a drug-drug salt with the antimicrobial agent proflavine (PF) as its proflavinium cation (HPF^+^), namely [HPF]_3_[Ga(ox)_3_]·4H_2_O, is reported together with the preparation of the potassium salt K_3_[Ga(ox)_3_] and the novel dimeric gallium(III) salt K_4_[Ga_2_(ox)_4_(μ-OH)_2_]·2H_2_O. All compounds have been characterized by solid state methods, and their performance as antimicrobial agents has been evaluated by disk diffusion assay against the bacteria strains *Pseudomonas aeruginosa* ATCC27853, *Staphylococcus aureus* ATCC25923, and *Escherichia coli* ATCC25922. While the [HPF]_3_[Ga(ox)_3_]·4H_2_O drug-drug salt is effective against all three strains, the gallium oxalate salt K_3_[Ga(ox)_3_] showed impressive selectivity towards *P. aeruginosa,* with little to no antimicrobial activity against the other two organisms. This work presents novel breakthroughs towards Ga based antimicrobial agents.

## Introduction

An area of increasing interest is the possibility of obtaining new materials with competitive physico-chemical and pharmaceutical properties via a combined crystal engineering^1^ and mechanochemical, solvent-free^2^ approach. More specifically co-crystallization methods based on the direct reaction in the solid state (or in slurry conditions) have proven to be well suited for the preparation of new compounds in the solid form for subsequent applications and uses in a variety of fields^3,4^.

One of these that is becoming increasingly important is that of microbe drug-resistance, i.e. the ability of bacteria to develop survival mechanism to evolve and protect themselves against the antimicrobial effect of pharmaceutical drugs, is increasingly affecting our ability to treat human and animal diseases^5–7^. Resistant microbes are difficult to treat leading us into the antimicrobial resistance (AMR) era^8^. Rather than increasing dosage, the possibility of finding alternative antimicrobial agents^9^, natural bacterial killers, or novel drug formulations are key to resolving this challenge^10^. Further there is a need for controlling spread via bacterial growth on objects, surfaces, tools, etc^11^. This led us to ask, could using metal complexes, with known or potential antimicrobial activity paired with conventional organic antimicrobials a way to treat drug-resistant pathogens?

Our objective towards contributing to the quest for novel antimicrobials relies on an approach used successfully in previous occasions to prepare compounds possessing enzymatic inhibition capacity against enzymes such as amino-mono oxygenase and urease^12,13^. Essentially, this approach utilizes crystal engineering methods, such as the direct reaction between preformed molecular salts, to combine organic biocides with metal atom complexes that also possess antimicrobial properties. With this idea in mind we have already synthesized, structurally characterized, and subjected to antimicrobial assays a series of compounds where the biocide proflavine is co-crystallized together with Ag, Cu, and Zn salts or metal complexes^14,15^, as all these metals exhibit strong antimicrobial activity^16,17^.

**Figure 1:**
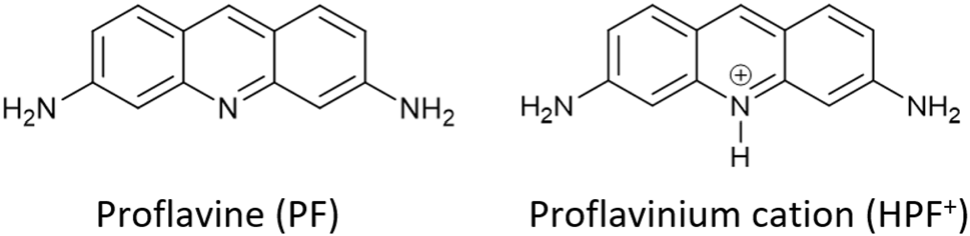
Neutral proflavine PF and monoprotonated proflavine, i.e. the proflavinium cation HPF^+^ used in this work.

Proflavine (see Fig. 1) is an established antimicrobial and is classified with the quaternary ammonium compounds (QCCs), which find use as antiseptics, cationic surfactants, disinfectants, herbicides, and dyes, with applications in domestic, clinical and industrial setting^18^. Proflavine antimicrobial mode of action is based on the intercalation between the nucleotide bases of DNA and further disrupting DNA replication and transcription^19^. Regions of DNA are also photosensitized upon intercalation and are subject to DNA strand breaks and point mutations^20^.

In this paper we extend our approach to encompass a metal atom, gallium, whose antimicrobial properties have begun to be investigated in recent times^21,22^. Gallium, in its complexes and salts, is an emerging metal-based antimicrobials whose mechanism of action is based on Fe atom mimicry and thus interference with enzymatic processes dependent on Fe. Ga is competitively incorporated in Fe-containing metalloproteins but does not readily undergo redox reactions under the physiological conditions and therefore leaves the iron-dependent enzymes unfunctional^23^. Ga ions can easily get into bacterial cells by exploiting the Fe uptake system through replacing the Fe in iron savaging siderophores, which has led Ga to be referred to as a “Trojan horse” antimicrobial.

Hereafter we report on the preparation, structural characterization, and evaluation of the novel oxalate gallium complexes K_3_[Ga(ox)_3_]·3H_2_O and K_4_[Ga_2_(ox)_4_(μ-OH)_2_]·2H_2_O and of the proflavinium adduct [HPF]_3_[Ga(ox)_3_]·4H_2_O. The antimicrobial performance of these novel compounds against the bacteria strains *Pseudomonas aeruginosa* ATCC27853, *Staphylococcus aureus* ATCC25923, and *Escherichia coli* ATCC25922 using the established antimicrobial disk diffusion assay has been investigated and the results described in the following.

## Materials and methods

All reagents and solvents were purchased from Sigma Aldrich and used without further purification.

### Synthesis of K_3_[Ga(ox)_3_]·3H_2_O

*Solution synthesis* Ga(NO_3_)_3_·9H_2_O (104.43 mg; 0.25 mmol) was dissolved in distilled water, and the solution was added to ca. 25 mL of an aqueous solution of K_2_C_2_O_4_·H_2_O (138.16 mg; 0.75 mmol). By addition of EtOH as antisolvent, a white suspension of K_3_[Ga(ox)_3_]·3H_2_O was obtained, which was centrifuged and left to dry at ambient conditions. Single crystals of K_3_[Ga(ox)_3_]·3H_2_O suitable for X-ray diffraction were grown in one week by vapour diffusion of EtOH into an aqueous solution containing Ga(NO_3_)_3_·9H_2_O and K_2_C_2_O_4_·H_2_O in 1:3 stoichiometric ratio. *Solid state synthesis* K_3_[Ga(ox)_3_]·3H_2_O could also be obtained in the solid-state, either by manual grinding in agata or porcelain mortar and via mechanical milling with a Retsch MM200 Mixer Mill. In this last procedure, the same quantities of solid reagents used for the solution synthesis were added to a 5 mL agate jar containing 2 balls of 5 mm diameter; the solid mixture was milled for 1 h, yielding a white fine powder of K_3_[Ga(ox)_3_]·3H_2_O (see SI for X-ray characterization).

### Synthesis of K_4_[Ga_2_(ox)_4_(μ-OH)_2_]·2H_2_O

A serendipitous result was obtained when a 1:3 stoichiometric mixture of Ga(NO_3_)_3_·9H_2_O (104.43 mg; 0.25 mmol) and K_2_C_2_O_4_·H_2_O (138.16 mg, 0.75 mmol) was dissolved in water, and acetone vapours were let to diffuse into the solution (vapor diffusion technique): at the aqueous solution/acetone interface single crystals formed in a matter of days, which were characterized via X-ray diffraction as the salt K_4_[Ga_2_(ox)_4_(μ-OH)_2_]·2H_2_O, containing the dimeric gallium(III) anion [Ga_2_(ox)_4_(μ-OH)_2_]^4−^ (see SI for a comparison of the X-ray experimental pattern and the one calculated on the basis of single crystal data).

### Synthesis of [HPF]Cl

Commercial [HPF]_2_[SO_4_]·xH_2_O (1 g) was dissolved in 25 mL of water and NaOH (3.10 g, 0.077 mol) was added to the solution, yielding PF·H_2_O^24,25^ as a crystalline powder. PF·H_2_O was then dissolved in an equimolar quantity of HCl 0.1 M under stirring at ambient conditions. The chloride salt was recovered after solvent evaporation, and its XRPD pattern compared with the one calculated from single crystal data^26^.

### Synthesis of [HPF]_3_[Ga(ox)_3_]·4H_2_O

A 3:1 stoichiometric mixture of PF·HCl·2H_2_O (281.708 mg, 1 mmol) and K_3_[Ga(ox)_3_]·3H_2_O (168.38 mg, 0.33 mmol) was dissolved in 35 mL of water; the solution was heated to 70 °C and left under stirring for 24 h. Upon solvent evaporation dark red crystals suitable for X-ray diffraction were obtained, which were filtered and left to dry at ambient conditions.

### Powder X-Ray diffraction

For phase identification purposes room temperature X-ray diffraction patterns were collected on a PANalytical X’Pert PRO automated diffractometer equipped with an X’celerator detector in the 2θ range 3°–40° (step size 0.0167, time/step 50 s, VxA 40 × 40).

### Single crystal X-ray diffraction

Single Crystal data for all compounds were collected at room temperature with an Oxford Diffraction X´Calibur equipped with a graphite monochromator and a CCD detector. Mo-Kα radiation (λ = 0.71073 Å) was used. Relevant crystal data and details of measurements are listed in Table S1. The structure was solved by the Intrinsic Phasing methods and refined by least squares methods against F^2^ using SHELXT-2016^27^ and SHELXL-2018^28^ with Olex2 interface^29^. Non-hydrogen atoms were refined anisotropically. H_CH_ atoms were added in calculated positions; H_OH_ and H_NH_ atoms were either located from a Fourier map or added in calculated positions and refined riding on their respective carbon, nitrogen, or oxygen atoms. One proflavinium cation was found to be disordered ca. 50:50 over two positions. Mercury 4.3^30^ was used for graphical representations and for powder patterns simulation on the basis of single crystal data. Crystal data can be obtained free of charge from the Cambridge Crystallographic Data Centre via https://www.ccdc.cam.ac.uk and have been allocated the accession numbers CCDC 2123788-2123789.

### Thermogravimetric analysis (TGA)

TGA measurements were performed using a Perkin-Elmer TGA7 under an N_2_ gas flow, at a heating rate of 5 °C min^−1^.

### Hot stage and cross polarized optical microscopy (HS-CPOM)

For a thermal characterization of the samples a Linkam TMS94 device, connected to a Linkam LTS350 platinum plate and supplied with polarizing filters, was used. The imaging software VisiCam Analyzer, from an Olympus BX41 stereomicroscope, was simultaneously employed.

### Antimicrobial activity

Antimicrobial efficacy was assessed as in previous work^14,15^ according to current disk diffusion susceptibility testing protocols^31^. *Pseudomonas aeruginosa* ATCC27853, *Staphylococcus aureus* ATCC25923, *Escherichia coli* ATCC25922 indicator strains were used in this study. All culturing was performed in Lysogeny Broth (LB) prepared in distilled water with 10 g/L NaCl (VWR International Co., Mississauga, Canada), 5 g/L yeast extract (EMD Chemicals Inc., Darmstadt, Germany) and 10 g/L tryptone (VWR Chemicals LLC, Solon, USA). Agar medium for disk diffusion assays was obtained by adding 15 g/L bacteriological agar (VWR International LLC, Solon, USA) to the above-mentioned LB. In an aseptic environment, 200 µl of overnight culture *of S. aureus*, *P. aeruginosa* or *E. coli* was spread on LB agar plates (2 plates per organism for two technical replicates) and left to dry at room temperature for 1 h. Stocks of 24 mg/mL stock solutions of single components and suspensions/slurries of target crystal compounds were prepared from powder using the LB media. Six antimicrobial disks (Oxoid Ltd, Basingstoke, UK) were placed into a vial with 300 µL of compound stocks (AgNO_3_, proflavine, and test compounds) and left to soak for 30 min with mixing every 10 min by inversion of the vial. Then disks were transferred to the plates (total of 2 replicates per compound per organism) and plates incubated at 37 °C for 24 h. The zone of growth inhibition was measured by a ruler. For one to see a zone of inhibition, the compounds need to release from the filter disk and diffuse through the LB-agar media a consequence of which gives a concentration gradient. Biocidal versus inhibitory effect was established by touching the inhibited growth zone with a microbiological loop and streaking to a fresh agar plate, if the growth appeared in this plate, the compound was considered bacteriostatic, if no growth was present, the compound was considered bactericidal. The ability of the compound to kill pre-formed bacterial lawn was performed as follows. In aseptic environment, 200 µL of overnight culture of *S. aureus*, *P. aeruginosa* or *E. coli* was spread on LB agar plates (2 plates per organism for replicates) and cultivated overnight to receive the bacterial lawn. Antimicrobial disks used for previously described disk diffusion assay were removed from the previous plate with sterile tweezers and placed upside-down on the plates with the pre-formed bacterial lawn. Plates were then left overnight at 37 °C and disks removed from the plates with sterile tweezers. Zones under the disks were then assessed for bacterial lysis.

## Results and discussion

**Figure 2:**
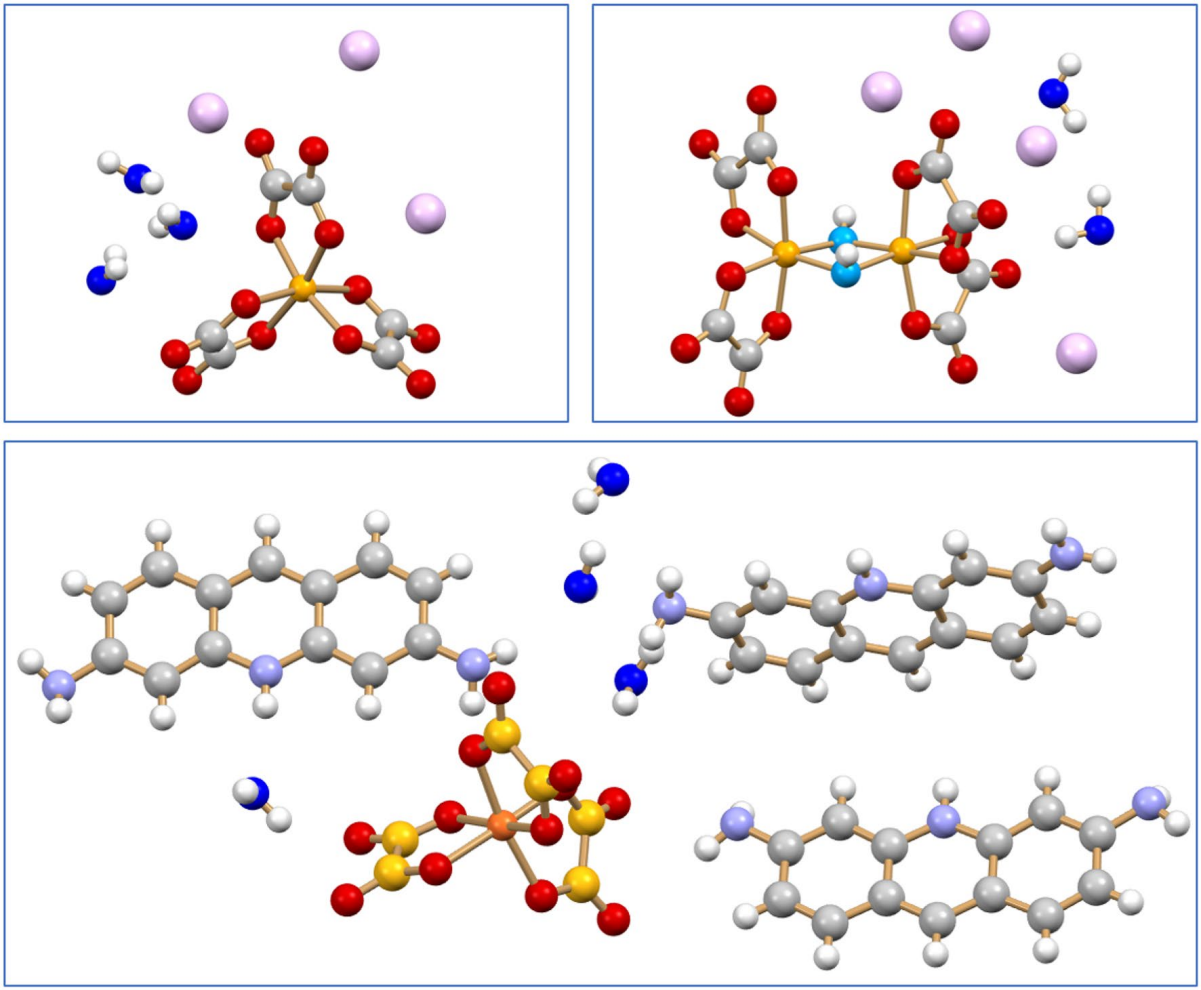
Schematic representation of the three compounds described in this work: K_3_[Ga(ox)_3_]·3H_2_O (top left), K_4_[Ga_2_(ox)_4_(μ-OH)_2_]·2H_2_O (top right) and the proflavinium adduct [HPF]_3_[Ga(ox)_3_]·4H_2_O (bottom). [K^+^ in violet, O_W_ atoms in blue, C_OX_ in light orange in the proflavinium adduct].

Before discussing the antimicrobial activity, it is worth examining the molecular and crystal structures of the three novel compounds K_3_[Ga(ox)_3_]·3H_2_O, K_4_[Ga_2_(ox)_4_(μ-OH)_2_]·2H_2_O and of the proflavinium adduct [HPF]_3_[Ga(ox)_3_]·4H_2_O (see Fig. 2).

**Figure 3:**
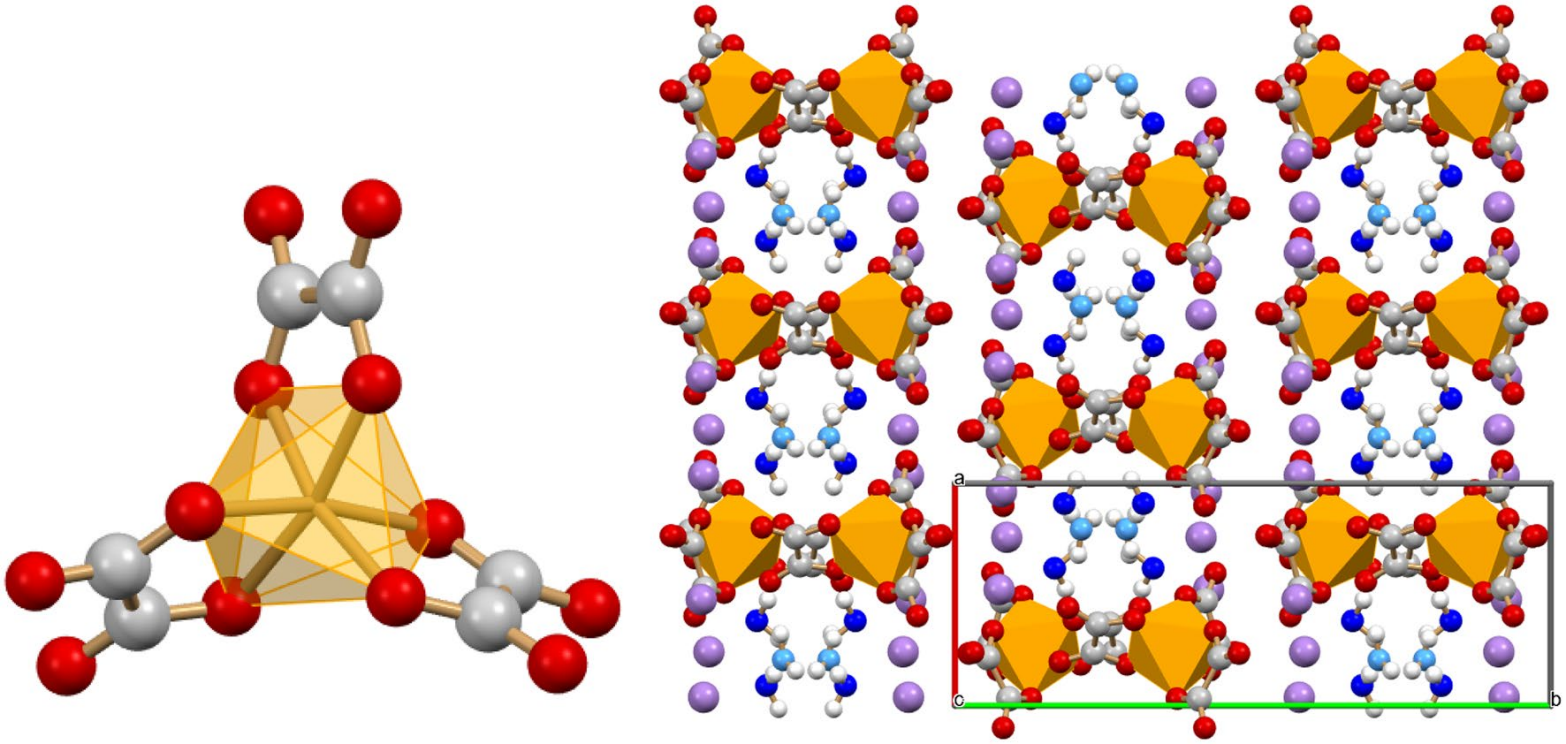
(Left) The structure of the [Ga(ox)_3_]^3−^ complex; (right) the channels accommodating the water molecules in the crystal structure of K_3_[Ga(ox)_3_]·3H_2_O. K^+^ in violet, O_W_ atoms in blue and cyano.

The trianion [Ga(ox)_3_]^3−^ in the salt K_3_[Ga(ox)_3_]·3H_2_O is isostructural with octahedral oxalate complexes of di- and trivalent metals, such as zinc(II), chromium(III), iron(III), aluminum(III), among others. The three oxalate dianions act as bidentate ligands towards gallium(III); the oxygen atoms not involved in the coordination to gallium interact in turn with the potassium cation. The first coordination sphere around the potassium cation is completed by water molecules that occupy channels extending along the crystallographic *c-*axis (see Fig. 3, left) and interacting via hydrogen bonds with the oxalate anions*.* TGA measurements (see Fig. S5) show the loss of only one water molecule per formula unit, most likely the one evidenced in azure in Fig. 3 (right), i.e. the water molecule located in the inner part of the channel.

**Figure 4:**
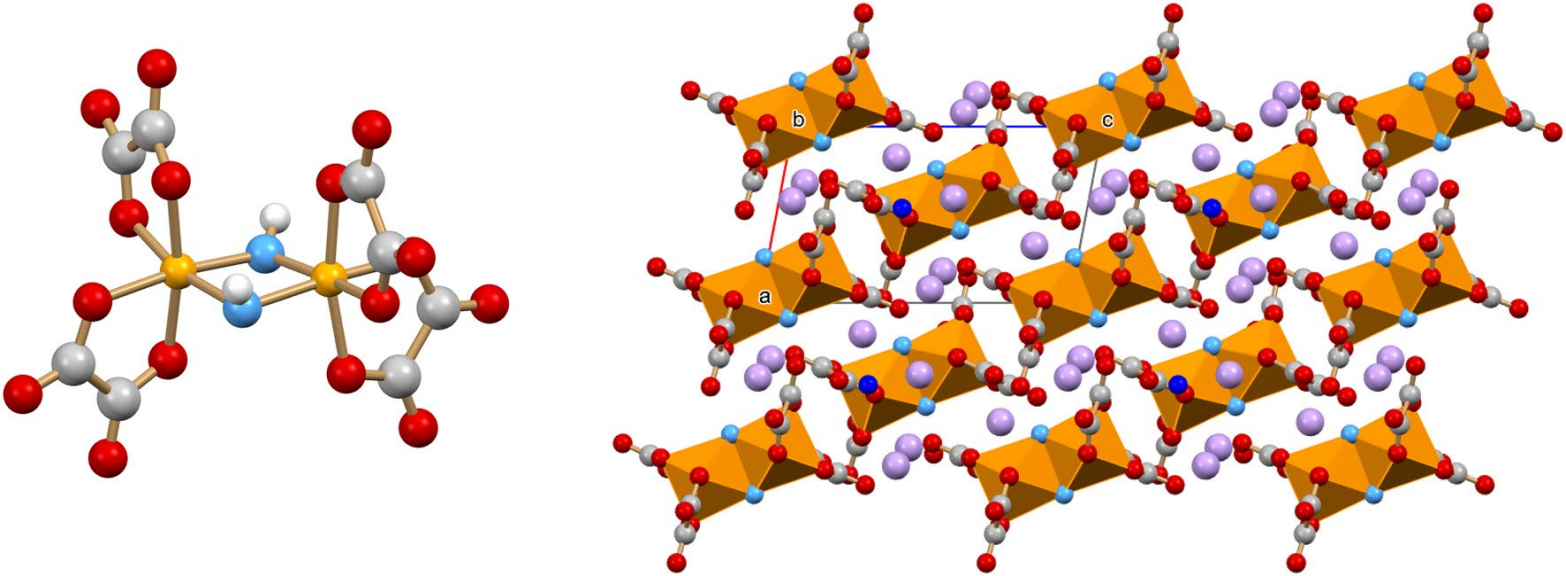
(Left) The [Ga_2_(ox)_4_(μ-OH)_2_]^4−^ dimeric anion showing the bridging OH groups; (right) the packing arrangement viewed along the *b*-axis in crystalline K_4_[Ga_2_(ox)_4_(μ-OH)_2_]·2H_2_O. K^+^ in violet, O_W_ in blue, O_OH_ in cyano; H atoms omitted for clarity.

The structure of K_4_[Ga_2_(ox)_4_(μ-OH)_2_]·2H_2_O is shown in Fig. 4. Each gallium(III) belonging to the dimeric anion is coordinated by two oxalate dianions and two bridging hydroxyl groups, with the six oxygen atoms describing a distorted octahedron around the metal centre. Ga-O_OH_ distances, in the range 1.929(6)–1.957(6) Å, are in line with the bond lengths found in the Cambridge Structural Database for related compounds.

**Figure 5:**
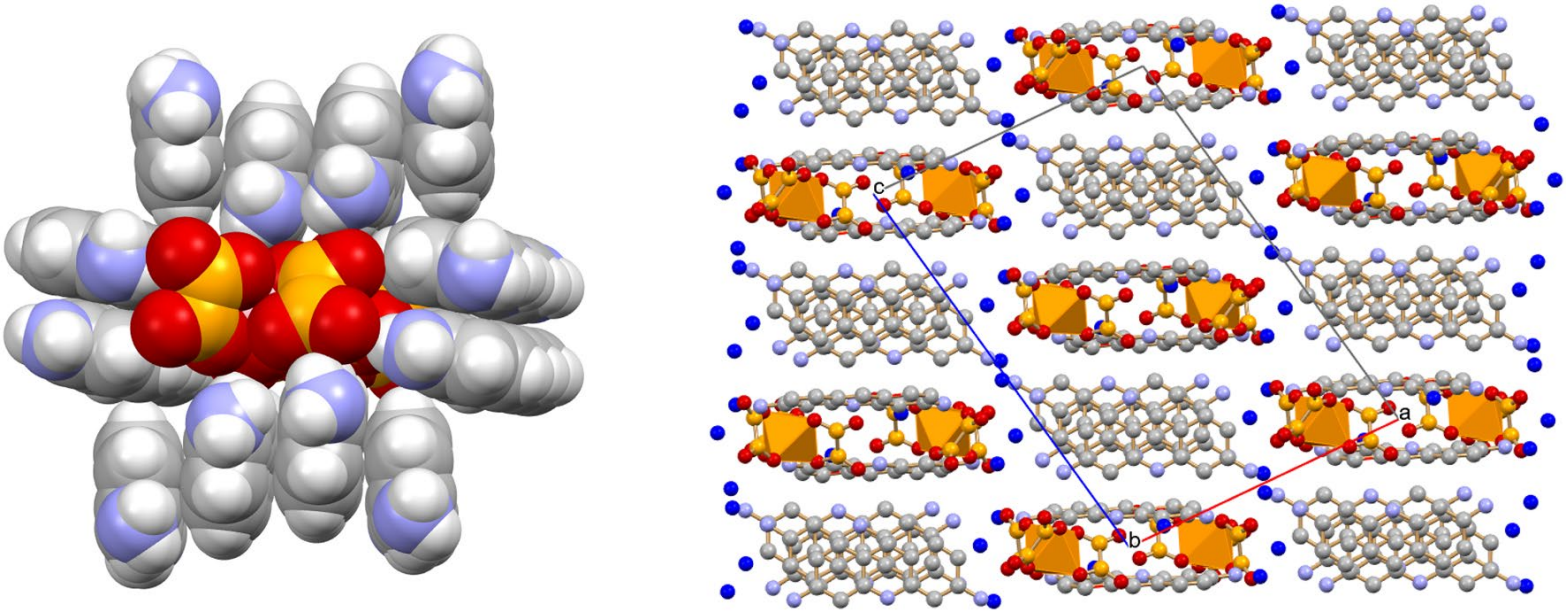
(Left) The proflavinium cations enveloping the [Ga(ox)_3_]^3–^ anions in crystalline [HPF]_3_[Ga(ox)_3_]·4H_2_O; (right) the packing in crystalline [HPF]_3_[Ga(ox)_3_]·4H_2_O viewed along the crystallographic *b*-axis. O_W_ in blue; H atoms omitted for clarity.

The structure of the proflavinium adduct [HPF]_3_[Ga(ox)_3_]·4H_2_O is shown in Fig. 5 (left). It is noteworthy that the proflavinium cations form stacks around the gallium tris oxalate core, in such a way as to completely envelope the anion. Figure 5 (right) shows the layered arrangement of the cations and the location of the water molecules, mostly arranged in layers parallel to the *b-*axis.

TGA measurement on [HPF]_3_[Ga(ox)_3_]·4H_2_O shows a loss of ca. 7% of the total molecular weight (see Fig. S6). Since the mass loss corresponds approximately to the percentage of water contained in the hydrated compound, it is likely due to complete dehydration. This process was also investigated via HSM. Single crystals of [HPF]_3_[Ga(ox)_3_]·4H_2_O were immersed in oil and heated at 10 °C min^−1^ from RT to 120 °C; the loss of water could be visually observed in the form of gaseous water bubbles starting at ca. 75 °C (see Fig. S7)*.* The dehydration process is reversible: variable temperature X-ray diffraction on powder (see Fig. S8) shows a different pattern for the anhydrous compound at 120 °C, but on going back to room temperature the water molecules are captured again, and the crystalline material recovered at the end of the heating–cooling cycle is the hydrated compound [HPF]_3_[Ga(ox)_3_]·4H_2_O.

## Antimicrobial assays

As found previously^32–34^, differences in antimicrobial susceptibility towards metal-based antimicrobials is seen between the three different strains. The strains chosen for evaluation include a Gram-negative enteric pathogen *Escherichia coli,* the opportunistic pathogen and ubiquitous soil organism *Pseudomonas aeruginosa* and the Gram-positive opportunistic pathogen *Staphylococcus aureus*. All three of these organisms are listed by World Health Organization as priority pathogens (*P. aeruginosa* and *E. coli*—critical priority, *S. aureus*—high priority), for which development of new antimicrobials is critically important^35^.

**Figure 6:**
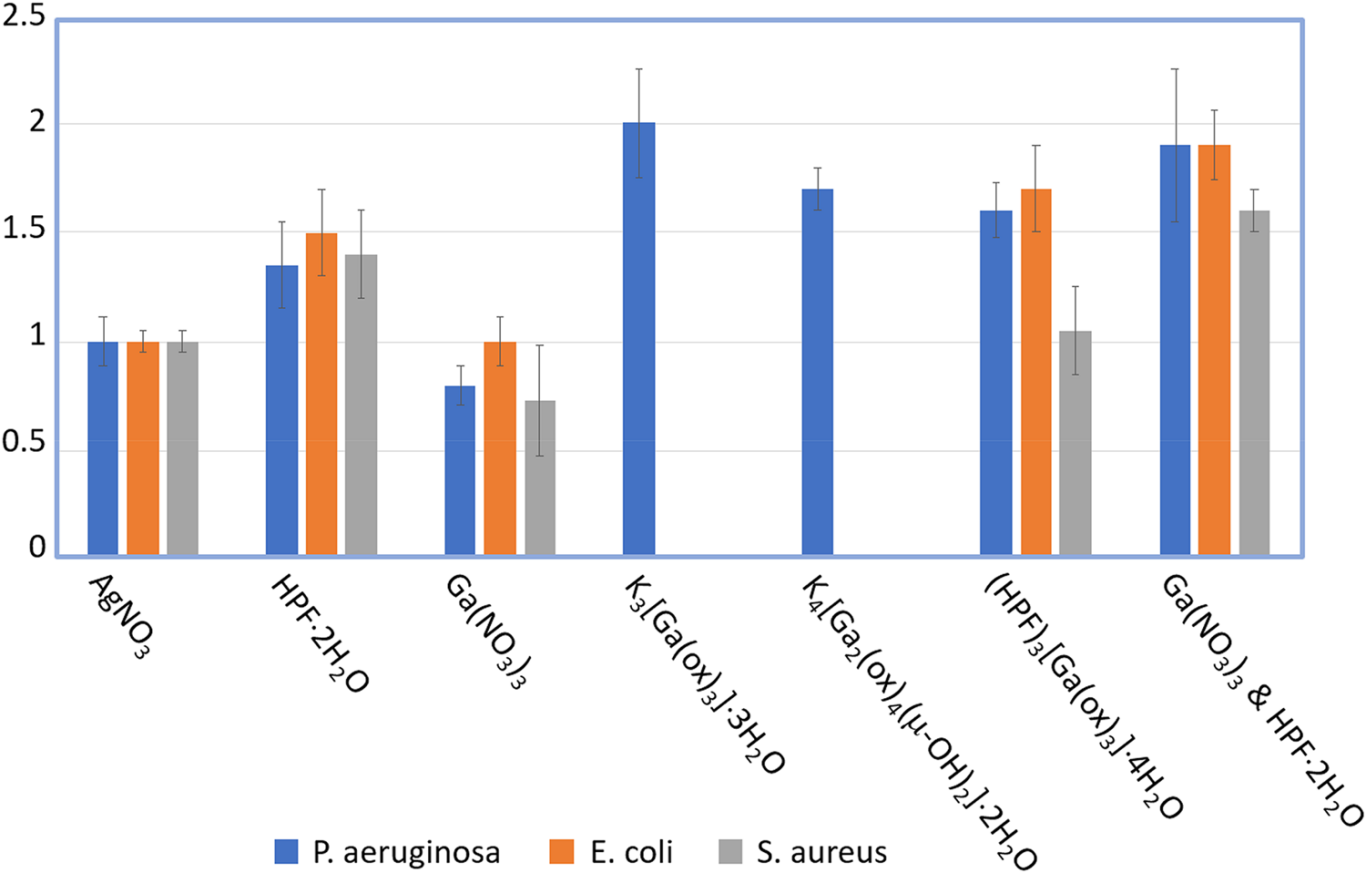
Antimicrobial efficacy of gallium compounds in comparison with proflavine and silver nitrate. The bars represent antimicrobial zone of inhibition assay where data are normalized to zone of AgNO_3_. The last set of columns on the right denotes effectiveness of combining the gallium nitrate salt with proflavine in solution both at 24 mg/mL, thus at higher molar load, and is designated by the ‘&’ notation. Data shows error deviations between trials. (n = 2–6 trials).

Antimicrobial activity was evaluated using the established antimicrobial disk zone^36,37^ of inhibition assay which has been used to explore similar proflavine–metal cocrystals^14,15^. Here we compare the zone of growth inhibition of bacterial strains spread on LB agar plates to that of silver nitrate. Silver is the most popular metal based antimicrobial^38^ and thus serves well to compare new metal based antimicrobial formulations to. Additionally, ratioing to the internal standard controls for plate-to-plate variations. Here, in Fig. 6, a value > 1 is more effective than silver and a value < 1 is less antimicrobial than silver.

We see that proflavine on its own has strong antimicrobial activity against all three strains, slightly greater than silver. K_3_[Ga(ox)_3_]·3H_2_O and K_4_[Ga_2_(ox)_4_(μ-OH)_2_]·2H_2_O behaved very similarly, showing efficacy only against *P. aeruginosa.* There was no visible zone of inhibition against either *E. coli* or *S. aureus.* It is somewhat remarkable to observe such selectivity of this formulation of Ga towards only the *Pseudomonas*, particularly since we see that Ga(NO_3_)_3_, which provides Ga^3+^ ions to enter via the Trojan horse mechanism, has activity slightly less than that of silver nitrate to all three organisms, as supported elsewhere^39^. In order to inflict toxicity to bacteria, Ga must be taken into the cell to replace Fe in proteins. The explanation likely originates through the difference of metallophore/siderophore acquisition systems for cellular iron uptake. *P. aeruginosa* primary system is through the *Pseudomonas* specific siderophore, pyoverdine^40^. Whereas *E. coli* uses a variety of diverse systems^41^, and *S. aureus* tends to take Fe from heme^42^. We can posit a hypothesis that the complex and dynamic structure of pyoverdine (consisting of a dihydroxyquinoline core, a 6–14 amino acid variable sequence peptide and a α-keto acid derived from the Krebs cycle) has the ability to coordinate the Ga-oxalate complex or outcompete the oxalate ligands for the Ga atom. Such dynamic metallophores are not produced by the other two bacteria. This reflects the different lifestyles and ecological niches of the investigated bacterial strains that leads to different siderophores available and thus differently evolved receptors and transporters^43^.

The [HPF]_3_[Ga(ox)_3_]·4H_2_O proflavine-gallium adduct showed efficacy against all three strains. The efficacy against *S. aureus* was the same as that of silver, but less than proflavine, but the combination worked well against the other two pathogens. To be noted, the co-crystal gave superior antimicrobial performance towards *E. coli* and *P. aeruginosa* essentially 50% better than proflavine alone. Here it suggests the presence of the gallium led to enhancement of proflavine toxicity to *E.* coli. To compare the crystal formulation, we loaded disks with a mixture of Ga(NO_3_)_3_ and HPF proflavine each at 24 mg/mL (last column set in Fig. 6). It required this very high load of antimicrobial mix to get to the same antimicrobial efficacy (within experimental variance) as the proflavine-gallium co-crystal ([HPF]_3_[Ga(ox)_3_]·4H_2_O, second last column in Fig. 6). If we consider the molar composition of the co-crystals, they contain less gallium and proflavine molecules per unit mass, thus their antimicrobial activity is likely much more superior than what is illustrated by the outcome presented in Fig. 6.

At this time, one can only provide conjectures to the mechanism of action of this drug-drug crystal. We can hypothesize that cell contact with the crystalline material induces changes in the physicochemical environment, leading to crystal decomposion on the cell surface. This would then provide a very high local dose of each of HPF and Ga ions for a devastating local ambush.

Assessment of contact killing properties of the compounds showed that all the novel compounds were able to lyse *E. coli* and *S. aureus* pre-grown lawn of cells upon overnight exposure, while the pre-grown *P. aeruginosa* cells were able to withstand the contact with the antimicrobial-soaked disks. This suggests that [HPF]_3_[Ga(ox)_3_]·4H_2_O, K_3_[Ga(ox)_3_]·3H_2_O and K_4_[Ga_2_(ox)_4_(μ-OH)_2_]·2H_2_O possess bactericidal properties with regard to *E. coli* and *S. aureus* but are bacteriostatic against *P. aeruginosa* in regard to a contact assay compared to the results of Fig. 6 where compounds are evaluated to inhibit growth.

## Conclusions

Metal-based antimicrobials have been gaining popularity as a new means to deal with the increasing antimicrobial resistance. The challenge is substantial and calls for the exploration of novel approaches and of novel antimicrobial agents. Gallium has not been as extensively explored as other metals in terms of antimicrobial activity per se and in conjunction with other molecules known to performance against common bacteria strains. In this paper we have reported the synthesis, structural characterization and antimicrobial performance of novel coordination compounds of gallium(III), namely the trianion [Ga(ox)_3_]^3−^ and the dimeric tetraanion [Ga_2_(ox)_4_(μ-OH)_2_]^4−^ as their hydrated K^+^ salts, and we have utilized the trianion [Ga(ox)_3_]^3−^ in the preparation of a co-crystal by reaction with the solid hydrochloride salt of the proflavinium cation HPF^+^. The product is a novel supramolecular salt containing three proflavinium cations in the form of the hydrated salt [HPF]_3_[Ga(ox)_3_]·4H_2_O. This latter compound has also been subjected to antimicrobial assay against ATCC pathogen indicator strains of *P. aeruginosa*, *S. aureus* and *E. coli*, and has been shown to be effective against all three strains, thus behaving as a drug-drug salt. This study also highlights specificity of gallium ligated species uptake between the three bacteria strains, with only *P. aeruginosa* able to recognize and uptake the gallium oxalate species.

## Supplementary Information

Supplementary Information.
