# Supplementary material for: Antimicrobial activity of supramolecular salts of gallium(III) and proflavine and the intriguing case of a trioxalate complex
Source: Sci Rep. 2022 Mar 7;12:3673. doi: 10.1038/s41598-022-07813-0 (PMC8901752; doi:10.1038/s41598-022-07813-0)
Supplement: Supplementary file 1 — Supplementary Information. [file 41598_2022_7813_MOESM1_ESM.docx]

**Antimicrobial activity of supramolecular salts of gallium(III) and proflavine and the intriguing case of a trioxalate complex**

**Marzia Guerrini, Simone d’Agostino, Fabrizia Grepioni, Dario Braga*
Dipartimento di Chimica “Giacomo Ciamician”, Università di Bologna, Via Selmi, 2 – 40126 Bologna – Italy.**

**Andrii Lekhan, Raymond J. Turner*
Department of Biological Sciences, University of Calgary, 2500 University Drive NW, Calgary, Alberta T2N 1N4, Canada**

**SUPPLEMENTARY INFORMATION**

**(6 pages)**

**1. Powder X-ray diffraction at room temperature............................................ page 2**

**2. Single crystal X-ray diffraction....................................................................... page 4**

**3. Thermal gravimetric analysis.......................................................................... page 5**

**4. Hot stage microscopy (HSM)........................................................................... page 6**

**5. Variable Temperature X-ray diffraction........................................................ page 6**

1. **Powder X-ray diffraction at room temperature**

**Figure S1.** Comparison of the experimental patterns for K_3_[Ga(ox)_3_]·3H_2_O obtained from solution (top, blue line) and from slurry (bottom, black line).

**Figure S2.** Comparison between the experimental pattern for the crystalline powder obtained from solution (top, blue line), and the calculated pattern for K_3_[Ga(ox)_3_]·3H_2_O (bottom, red line).

**Figure S3.** Comparison between the experimental and calculated patterns for the dimeric complex K_4_[Ga_2_(ox)_4_(μ-OH)_2_]·2H_2_O.

**Figure S4.** Comparison between experimental (top, black line) and calculated (bottom, blue line) diffraction patterns for [HPF]_3_[Ga(ox)_3_]·4H_2_O.

1. **Single crystal X-ray diffraction**

**Table S1.** Crystal data and details of measurement for K_3_[Ga(ox)_3_]⸱3H_2_O, K_2_[Ga_2_(ox)_4_(μ-OH)_2_]⸱2H_2_O and [HPF]_3_[Ga(Ox)_3_]⸱4H_2_O [common to all: T = 293 K, MoKα radiation (λ = 0.71073Å)]

| Formula | C_6_H_6_GaK_3_O_15_ | C_8_H_6_Ga_2_K_4_O_20_ | C_45_H_44_GaN_9_O_16_ |
| --- | --- | --- | --- |
| MW | 505.13 | 717.97 | 1036.61 |
| Crystal system | Monoclinic | Monoclinic | Monoclinic |
| Space group | P2_1_/c | Pn | P2_1_/n |
| a /Å | 7.7417(2) | 7.0084(3) | 15.2579(5) |
| b /Å | 19.7324(5) | 12.1280(4) | 13.7724(7) |
| c /Å | 10.3243(3) | 12.1257(5) | 22.2741(9) |
| α /° | 90 | 90 | 90 |
| β /° | 108.032(3) | 100.926(4) | 100.682(4) |
| γ /° | 90 | 90 | 90 |
| Volume /Å^3^ | 1499.70(7) | 1011.98(7) | 4599.5(3) |
| Z | 4 | 2 | 4 |
| ρ_calc_ /g cm^-3^ | 2.237 | 2.356 | 1.497 |
| μ /mm^‑1^ | 2.75 | 3.586 | 0.682 |
| F(000) | 1000 | 707.3 | 2144 |
| 2θ-range /° | 6.906 to 58.898 | 6.72 to 58.46 | 6.682 to 49.998 |
| Reflections collected | 6769 | 4742 | 17105 |
| Indep. refl.s | 3418 [R_int_ = 0.0268] | 2977 [R_int_ = 0.0283] | 8089 [Rint = 0.0420] |
| Data/restraints/param.s | 3418/6/252 | 2977/11/332 | 8089/748/797 |
| GOF on F^2^ | 1.057 | 1.053 | 1.079 |
| R1 [I>=2σ (I)] | 0.0397 | 0.0355 | 0.0783 |
| wR_2_ [all data] | 0.1120 | 0.0743 | 0.1647 |

1. **Thermal gravimetric analysis**


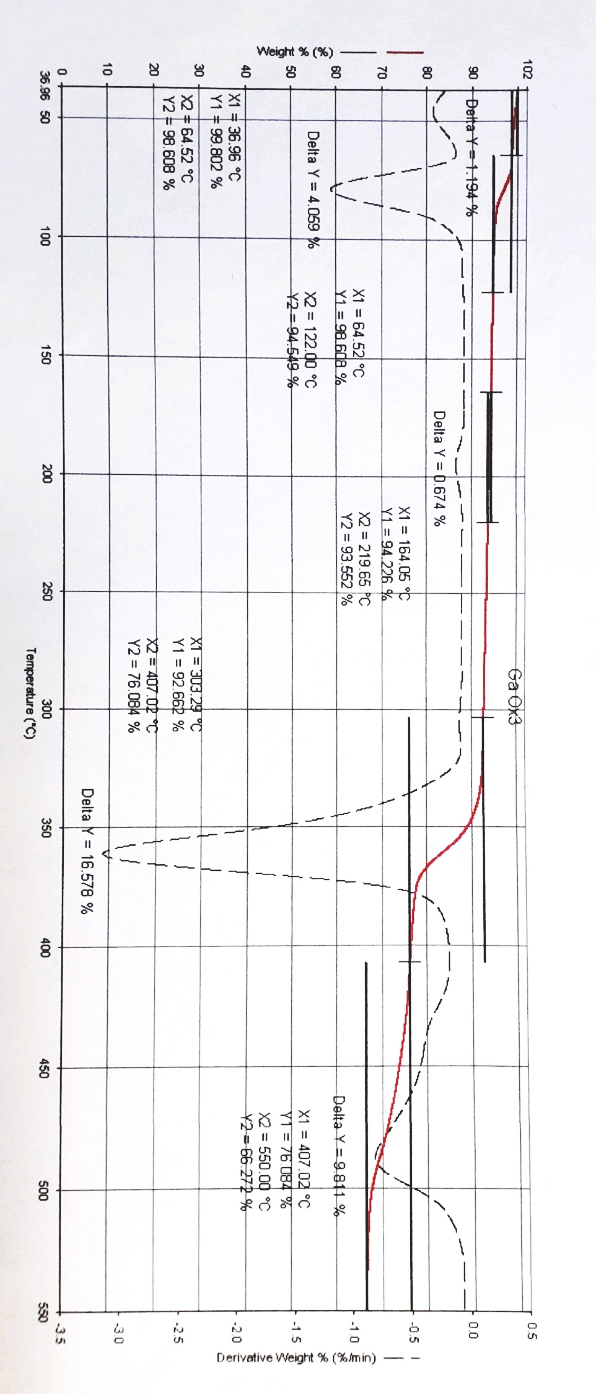


**Figure S5.** TGA trace for K_3_[Ga(ox)_3_]·4H_2_O.


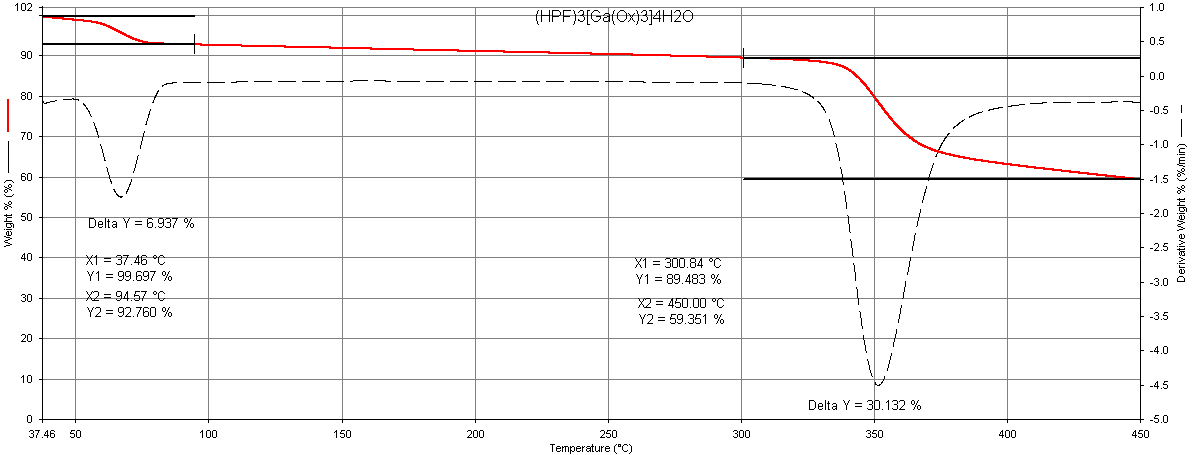


**Figure S6.** TGA trace for [HPF]_3_[Ga(ox)_3_]·4H_2_O

1. **Hot stage microscopy (HSM)**


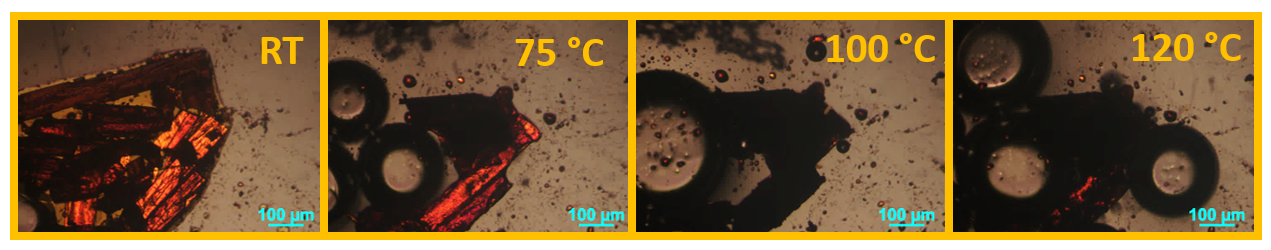


**Figure S7.** Hot stage microscopy images of [HPF]_3_[Ga(ox)_3_]·4H_2_O single crystals at different temperatures during the heating ramp at hot stage microscopy (from left to right: RT, 75°C, 100°C, 120°C)

1. **Variable Temperature X-ray diffraction**

**Figure S8.** X-ray diffraction patterns on crystalline [HPF]_3_[Ga(ox)_3_]·4H_2_O measured (bottom) at room temperature, (middle) at 120 °C and (top) back to room temperature. The pattern at 120 °C is markedly different from the hydrated form, and corresponds to an anhydrous phase, which transforms back to the hydrated compound once it is cooled to room temperature.
